# The sexual attitudes and lifestyles of London's Eastern Europeans (SALLEE Project): design and methods

**DOI:** 10.1186/1471-2458-9-399

**Published:** 2009-10-30

**Authors:** Alison R Evans, Violetta Parutis, Graham Hart, Catherine H Mercer, Christopher Gerry, Richard Mole, Rebecca S French, John Imrie, Fiona Burns

**Affiliations:** 1 Centre for Sexual Health and HIV Research, Research Department of Infection and Population Health, University College London, 3rd Floor Mortimer Market Centre, London WC1E 6JB, UK; 2 School of Slavonic and East European Studies, University College London, Gower Street, London WC1E 6BT, UK; 3 London School of Hygiene and Tropical Medicine, Keppel St, London WC1E 7HT, UK; 4 Africa Centre For Health and Population Sciences, University of KwaZulu-Natal, Mtubatuba 3935, South Africa; 5 National Centre in HIV Social Research - Australia, Robert Webster Building, The University of New South Wales, Sydney 2052, Australia

## Abstract

**Background:**

Since May 2004, ten Central and Eastern European (CEE) countries have joined the European Union, leading to a large influx of CEE migrants to the United Kingdom (UK). The SALLEE project (sexual attitudes and lifestyles of London's Eastern Europeans) set out to establish an understanding of the sexual lifestyles and reproductive health risks of CEE migrants. CEE nationals make up a small minority of the population resident in the UK with no sampling frame from which to select a probability sample. There is also difficulty estimating the socio-demographic and geographical distribution of the population. In addition, measuring self-reported sexual behaviour which is generally found to be problematic, may be compounded among people from a range of different cultural and linguistic backgrounds. This paper will describe the methods adopted by the SALLEE project to address these challenges.

**Methods:**

The research was undertaken using quantitative and qualitative methods: a cross-sectional survey of CEE migrants based on three convenience samples (recruited from community venues, sexual health clinics and from the Internet) and semi-structured in-depth interviews with a purposively selected sample of CEE migrants. A detailed social mapping exercise of the CEE community was conducted prior to commencement of the survey to identify places where CEE migrants could be recruited. A total of 3,005 respondents took part in the cross-sectional survey, including 2,276 respondents in the community sample, 357 in the clinic sample and 372 in the Internet sample. 40 in-depth qualitative interviews were undertaken with a range of individuals, as determined by the interview quota matrix.

**Discussion:**

The SALLEE project has benefited from using quantitative research to provide generalisable data on a range of variables and qualitative research to add in-depth understanding and interpretation. The social mapping exercise successfully located a large number of CEE migrants for the community sample and is recommended for other migrant populations, especially when little or no official data are available for this purpose. The project has collected timely data that will help us to understand the sexual lifestyles, reproductive health risks and health service needs of CEE communities in the UK.

## Background

On 1st May 2004, eight Central and Eastern European (CEE) countries - the Czech Republic, Estonia, Hungary, Latvia, Lithuania, Poland, Slovakia and Slovenia - joined the European Union (EU). The United Kingdom (UK) granted relatively unrestricted work and residency rights to nationals from the "Accession 8" (A8) and subsequently experienced a large influx of predominantly young economic migrants from these countries. It is estimated that over one million A8 nationals have migrated to the UK since accession [1,2].

Romania and Bulgaria (A2) joined the EU on 1st January 2007. More stringent restrictions were imposed on A2 migrants which incorporate the requirement for job and person specific work permits. In spite of this, over 42,500 Romanians and 28,000 Bulgarians registered for National Insurance numbers between January 2007 and December 2008 [3]. Throughout the rest of this paper, we refer to A8 and A2 migrants as simply CEE migrants.

The CEE migrant population in the UK is potentially vulnerable to sexual ill-health and reproductive morbidity. The 1990s saw huge increases in sexually transmitted infection (STI) and HIV rates across Central and Eastern Europe [4] and despite subsequent declines and variations across the region, STI and HIV rates remain high [5,6]. In addition to the background prevalence of STIs and HIV, the demographic profile of CEE migrants indicates that they are likely to be sexually active and have reproductive ambitions (over 80% of those registered with WRS are aged 18-34) while the provision of sex education in their countries of origin is limited [7,8].

CEE nationals have entitlement to NHS services and it is important to ensure that the health needs of these communities are met. However, the uptake of safer sex measures and patterns of health service use among CEE migrants in the UK are unknown. The SALLEE project (Sexual Attitudes and Lifestyles of London's Eastern Europeans) set out to address the lack of published research on sexual and reproductive health among CEE migrants in the UK.

There are a number of challenges to researching sexual behaviour among migrant communities. CEE nationals make up a small minority of the population resident in the UK with no sampling frame from which to select a probability sample. Furthermore, the information needed to design a convenience sampling strategy for the CEE migrant population is limited. Many members of the population are recent arrivals; they are also likely to be highly mobile and may not register to work through official channels, adding to the difficulty of estimating the socio-demographic and geographical distribution of the population. In addition, measuring self-reported sexual behaviour which is generally found to be problematic [9], may be compounded among people from a range of different cultural and linguistic backgrounds. This paper will describe the methods adopted by the SALLEE project to address these challenges.

### Research question

The SALLEE project set out to establish an understanding of the sexual lifestyles, reproductive health risks and health service needs of the CEE communities in London in order to inform service planning and the development of culturally appropriate health promotion and HIV prevention material.

The specific research objectives were to:

• conduct detailed social mapping of CEE community venues

• describe the sexual behaviour and attitudes, sexual and reproductive health and health service use of this population

• identify CEE migrants' specific sexual and reproductive health needs that are appropriate intervention targets through interdisciplinary qualitative research investigating culture, beliefs, practices, expectations and social, health and sexual behaviours, including aspects of relationships

• evaluate the differences in population recruited by the different sampling methods

The project was funded by the UK Medical Research Council (2007 - 2009) and conducted by researchers from the Centre for Sexual Health and HIV Research and the School of Slavonic and Eastern European Studies at University College London. An Expert Advisory Group consisting of people with academic expertise in the areas of sexual health and migration was set up to advise on the design and development of the study. A Community Advisory Group consisting of representatives from each of the ten CEE countries was set up in order to review the development of the study and ensure that the study design was appropriate and acceptable to the target population.

### Ethics

The research protocol was approved by the Camden and Islington Research Ethics Committee.

## Methods/Design

The project consisted of a quantitative arm and a qualitative arm: a cross-sectional survey of CEE migrants, augmented with semi-structured in-depth interviews with a purposively selected sample of CEE migrants. The principal aim of the cross-sectional survey was to provide generalisable data on sexual and reproductive health, risk behaviours and health service use of CEE migrants in London. The qualitative research was designed to complement the quantitative survey by further exploring these issues and identifying additional important elements.

This account of the design and methods of the SALLEE project will describe the survey instrument, sampling approach, recruitment process, sample characteristics and data analysis for the quantitative arm. This will be followed by a description of sampling, recruitment, data collection, data analysis and sample characteristics for the qualitative arm.

### Quantitative arm

#### Survey instrument

The survey instrument was developed in consultation with members of the Expert and Community Advisory Groups. It was a self-completed questionnaire that was fielded using hand-held computers for the community and sexual health clinic samples and a web survey for the Internet sample (the composition and recruitment of these samples is described below). Each version of the questionnaire contained exactly the same question wording. The questionnaire was confidential and anonymous, including no information that would allow respondents to be identified.

Where possible, questions were taken from Natsal, the British National Survey of Sexual Attitudes and Lifestyles [10] or other previously validated questionnaires (such as the British Labour Force Survey) in order to maximise their reliability and validity. The questionnaire was piloted with nine CEE migrants to examine its feasibility and acceptability and to explore understanding of the question items and underlying constructs. The use of the hand-held computers and the questionnaire routing were also tested during this phase of piloting. The questionnaire was modified in the light of this feedback, and then sixteen people tested the web survey to check question routing and technical usability. The questionnaire was translated into eleven languages (the ten languages of the CEE countries plus Russian) and the translation accuracy was checked by other bilingual native speakers of the eleven languages.

The final questionnaire took about ten minutes to complete. It contained a total of 160 questions though many of these were specifically worded for men or women or were skipped depending on how the respondent answered particular questions. All but one of the items (home post-code) required respondents to tick a box or fill in a number. Open questions were avoided because respondents often provide inadequate answers to open questions in self-administered questionnaires [11].

In addition to information sheets, provided for the community and clinic samples, the questionnaire was preceded by a summary of the content and nature of the questions. Respondents were then asked to tick a box if they agreed to take part, which indicated their consent. The first section of the questionnaire asked detailed background information, including socio-demographic characteristics and use of GP services. The main section concentrated on sexual and reproductive health, including sexual practices, numbers of partnerships, use of condoms, paying for sex, abortion history, STIs and HIV, and use of sexual and reproductive health services. Finally, respondents were asked about their use of alcohol and recreational drugs and their attitudes towards sexual and reproductive behaviours.

#### Sampling

As a new migrant population, there is no adequate sampling frame from which to draw a probability sample of CEE nationals in London. The study therefore relied on convenience sampling in order to generate a cost effective sample which would be large enough for detailed analysis. As all convenience methods have their own strengths and weaknesses and are subject to bias, this study adopted three convenience sampling strategies (community, clinic and Internet) in order to ensure representation of elements of the population which were key to the research question and triangulate methods.

##### Community sample

The community sample aimed to generate a representative cross-section of CEE migrants in London. Greater London has a population of about 7.6 million and covers an area of around 1,600 km^2 ^[12]. As CEE migrants represent a minority of the population in London and there is limited information on their geographical distribution, we undertook a process of social mapping to identify where CEE nationals were likely to be found. In the absence of a single complete data source on CEE migrants in London, we used data from a number of sources to estimate the numbers CEE migrants in each of the 33 London boroughs:

• The Labour Force Survey (LFS) is a quarterly sample survey of households living at private addresses in Great Britain, excluding students in halls who do not have a UK resident parent and people in most other types of communal establishments [13]. The median number of CEE people living in the London boroughs according to the LFS was 2,500 (range = 1,000 to 16,000).

• Data from the Electoral Register were provided by 22 of the 33 London boroughs at the request of the research team. The data include anyone who is a CEE national who has registered to vote as a resident of each borough. It may be biased towards CEE people who are likely to settle for longer periods in the UK. The median number of CEE people living in the London boroughs according to the Electoral Register was 2,273 (range = 39 to 11,183).

• National Insurance Number (NINO) Registrations include anyone who has registered for a NINO in order to work or claim benefits in the UK, using their most recently recorded address [3]. A cumulative total of NINO registrations for CEE nationals over the years 2003 - 2007 for each borough was calculated. The median number of CEE people living in the London boroughs according to the NINO Registrations was 3,840 (range = 70 to 17,960).

• The Worker Registration Scheme (WRS) provides data on the socio-demographic characteristics, employment sector and employer location for A8 workers registering to work in the UK [1,14]. Since accession, people from the A8 countries have been required to register under the WRS in order to work for an employer in the UK for more than one month. This is the only source of data on where A8 people work but it excludes A8 students and the self-employed, as well as A2 migrants. The median number of A8 people working in the London boroughs according to the WRS was 1,608 (range = 319 to 14,466).

We also asked members of the CEE community about the areas of London where CEE migrants were most likely to live. We consulted our Community Advisory Group and posted a web survey on the Internet asking people to identify venues in London used by Central and Eastern Europeans. The url link to the survey was sent to students and staff at the School of Slavonic and Eastern European Studies, UCL and other London Universities.

The score on the Indices of Deprivation [15] was used to assess the level of deprivation in each of the boroughs. The median index was 25.0 (range = 9.6 to 46.1: least to most deprived).

Data collection was limited to two London boroughs due to financial and logistical constraints. Both data and community sources suggested Newham which is in London's East End and Hammersmith & Fulham which is in the West of the capital. Newham is the London borough with the highest number of CEE residents according to the LFS (16,000), second highest number of NINO registrations (14,220) and an average number of A8 nationals (1,332) registered to work. It also has one of the highest levels of deprivation in London (43.0) and has a long history of settlement by migrants, including those from Central and Eastern Europe.

Hammersmith & Fulham has been traditionally settled by Polish migrants. It has the sixth highest number of A8 people working there, according to the WRS (3,558) and the number of CEE people living there is about average, according to the LFS (2,000), Electoral Register (2,926) and NINO registrations (5,070). It has a medium level of deprivation (28.1).

A detailed community mapping exercise was undertaken in order to identify places where fieldworkers would be able to approach potential respondents. Members of the research team walked the streets of Newham and Hammersmith & Fulham looking for suitable venues. We used information sources, such as community and ethnic organisation websites, to identify other possible venues and events. In order not to over-use successful venues and to replace unsuccessful ones, we continued to identify new venues and events over the data collection period. A total of 82 venues and events were located, including supermarkets, high street shops, Eastern European shops, schools, restaurants, toddler groups, tube stations, a Czech film season (November 2008) and a Lithuanian Christmas Fair (December 2008).

##### Clinic sample

The clinic sample aimed to ensure representation of sexual health service users within the study. The evidence also suggests that clinic samples capture those with higher risk behaviour [16]. Respondents were recruited from patients at two GUM (Genito-Urinary Medicine) clinics and one family planning clinic in North and Central London.

##### Internet sample

The Internet sample enabled participation of CEE people in London who may not otherwise be included in the study due to the geographic constraints of the other samples. It may also have encouraged people who prefer complete anonymity to take part.

#### Recruitment

The primary outcome measures were one or more new sexual partners in the past year (a measure of high-risk sexual behaviour) and whether the respondent had a GP (a measure of health service utilisation). Previous studies suggest that approximately 25% of people aged 16-44 in Britain have had one or more new sexual partners in the past year [10], and approximately 75% of migrant populations have a GP [17]. We estimated that a community sample size of 2000 would allow prevalence estimates for either outcome with 95% confidence intervals, as well as adequately powered subgroup analysis, for example by gender, sexuality or geographical region. The greater assumed homogeneity of the clinic sample suggested a smaller sample of 350.

Eligible respondents were literate men and women who were aged 18 years or over and self-identified as migrants from one of the ten CEE countries. The project was described as a study of Central and Eastern Europeans in London and all respondents were asked to provide the first half of their home post-code. The community and clinic samples were both recruited in London and the web survey was advertised on websites for CEE nationals in London and the UK.

##### Community sample

Fieldwork took place over a nine month period (July 2008 - March 2009). The nine fieldworkers involved in the recruitment of respondents for the community sample were native speakers of six of the languages of the CEE countries. The positions were advertised on the UCL website, the UCL union website and at jobs.ac.uk. Fieldworkers undertook one day of training, covering the content of the questionnaire and administration of the survey with members of the research team. A monthly rota was drawn up whereby venues or events identified in the social mapping exercise were attended by pairs of fieldworkers in four to five hour shifts. Some venues proved unsuccessful because it turned out that they were not used very much by CEE nationals. Such venues were excluded from future rotas. Attendance at more successful venues was deliberately varied by day and time of day. During each shift, fieldworkers approached every man and woman that they believed to be a CEE migrant. Fieldworkers made a note of the nationality, estimated age and gender of eligible respondents that they approached who refused to take part. All eligible respondents were given an information sheet about the study in their native language and were offered a £5 high street voucher as an incentive. The questionnaire was self-completed on hand-held computers and was available in the ten languages of the CEE countries, plus Russian and English. When they had completed the questionnaire, respondents were offered a leaflet containing a list of websites related to sexual health and health services in the UK.

Fieldworkers came in to the research office once a week to download the data from their hand-held computers. In addition to these regular one-to-one meetings, monthly group meetings were held with the fieldworkers to discuss the data collection process and administrative matters. The meetings provided useful feedback on data collection and helped to support and motivate the staff.

A total of 2,284 respondents completed the questionnaire in the community sample. Eight respondents were excluded because they did not appear to be CEE migrants on the basis of country of birth, passport(s) held and language spoken at home while growing up. This resulted in a total of 2,276 participants. Altogether, 6,781 adults were approached but 4,497 declined to take part, resulting in a response rate of 33.6%. There was no significant difference in the likelihood of participation according to gender or the borough where respondents were approached. Those who refused to take part were estimated to be slightly older (30.2 yrs vs 29.1 yrs, p < 0.01).

##### Clinic sample

The recruitment period for the clinic sample coincided with the community sample (July 2008 - March 2009). Recruitment took place in two sexual health clinics, the Mortimer Market Centre and the Archway Sexual Health Clinic, and one family planning clinic, the Margaret Pyke Centre, in North and Central London. The recruitment strategy changed over the period of data collection as more efficient methods were developed. After initially allocating fieldworkers to collect data in the clinics, we found that this was not an efficient use of their time because CEE patients represented a minority of patients at the clinics. A system was developed whereby researchers on the project used information provided by reception staff and the appointments database in order to determine when potential respondents arrived at the clinics. The days and times on which data collection took place in the clinics varied by clinic and over the data collection period. During the times that researchers undertook clinic recruitment, they approached every man and woman that was identified as a CEE migrant and that they were able to locate in the clinic. Patients were asked if they would be willing to complete the questionnaire on a hand-held computer using the same procedure as the community sample. They usually completed the questionnaire in the clinic waiting room while they were waiting for their appointment. They were also given a £5 incentive for taking part.

A total of 358 respondents completed the questionnaire in the clinic sample. One respondent was excluded because they did not appear to be a migrant from one of the CEE countries, resulting in a total of 357. Altogether, 387 CEE patients were approached but 30 declined to take part resulting in a response rate of 92.2%. There was no significant difference in the likelihood of participation according to age, gender or the clinic where respondents were approached

##### Internet sample

Internet recruitment took place from July 2008 to March 2009. The url link to the web survey was emailed to contacts in the CEE community and was placed on homepages of websites that were aimed at CEE communities in the UK or those seeking sexual or reproductive health services. No financial incentive was offered for taking part in the web survey. The School of Slavonic and Eastern European Studies distributed the link to its staff and students, and the survey was also advertised on websites aimed at CEE migrants in the UK http://www.pohyby.co.uk, http://www.labrit.co.uk, http://www.anglija.lt, http://www.pczs.org and people interested in sexual health services http://www.mariestopes.org.uk. We also targeted lesbians, gay men, bisexual and transgender (LGBT) people by placing the link on an LGBT university website http://www.metlgbt.co.uk. These websites placed links to the web survey upon request and we have no data on the exact time that the link was posted or taken down.

During this period a total of 487 web responses were submitted. The responses indicated that they were all CEE migrants. Just under a quarter (115/487; 23.6%) of respondents gave their home post-code as outside London. This results in a London Internet sample of 372 respondents. We do not know how many 'hits' the url link received, nor how many people started but did not complete or submit the web survey.

#### Sample characteristics and data analysis

A total of 3,005 eligible respondents took part in the SALLEE project. The background characteristics of respondents from the three samples are presented in Table 1. The mean age of all respondents was 28.8 years and the clinic sample was the youngest (26.9 yrs, p < 0.01). While half of the community and Internet samples were male (48.5% and 47.0%, respectively), only a quarter of the clinic sample was male (26.6%, p < 0.01). Three quarters of the community sample were born in A8 countries (rather than Bulgaria or Romania) whereas nearly all of the Internet sample were born in A8 countries (74.2% vs 97.8%, p < 0.01). The majority of all samples were employed but the clinic and Internet samples were more likely to be employed than the community sample (80.7% and 84.1% respectively vs 72.8%, p < 0.01). The community sample was also less likely to have completed higher education than the clinic and Internet samples (29.4% vs 53.9% and 43.9%, p < 0.01), more likely to be married or co-habiting (52.7% vs 42.0% and 47.3%, p < 0.01). The community and Internet samples were more likely than the clinic sample to have arrived in the UK since the A8 accession in May 2004 (79.5% and 78.1% vs 62.2%, p < 0.01).

**Table 1:** Background characteristics of London respondents in quantitative arm, by sample

	Community sample n = 2,276		Clinic sample n = 357		Internet sample n = 372		p value
	**n**	**%**	**n**	**%**	**n**	**%**	

Age (mean; sd)	29.1 (8.6)		26.9 (4.5)		28.3 (6.6)		< 0.01
Male	1,103	48.5	95	26.6	175	47.0	< 0.01
Born in A8 country	1,679	74.2	296	83.4	357	97.8	< 0.01
Working	1,647	72.8	288	80.7	313	84.1	< 0.01
Completed higher education	668	29.4	192	53.9	163	43.9	< 0.01
Married or co-habiting	1,189	52.7	150	42.0	172	47.3	< 0.01
Arrived in UK since May 2004	1,762	79.5	219	62.2	285	78.1	< 0.01

Data analysis will be undertaken using standard statistical packages to examine sexual and reproductive health, risk behaviours and health service use of CEE migrants in London. We will compare data from the three samples.

### Qualitative arm

#### Sampling

A purposively selected sample of 40 respondents was recruited over the data collection period. Participants were sampled to ensure a range of personal characteristics and experiences. The preliminary sampling criteria identified during the analysis of contextual and secondary data were gender, age group and time in the UK. The secondary criteria were region of origin, sexual orientation, use of sexual health services in the UK and partnership status (Table 2).

**Table 2:** Interview quota matrix

Primary quotas			
**Age**	**Male**	**Female**	**Total**
18-24	6-8	6-8	12-16
25-34	4-6	4-6	8-12
35-45	4-6	4-6	8-12
45+	4-6	4-6	8-12
**Residence in UK**			
<1 year	10-15		12-15
1-4 years	10-15		12-15
>4 years	10-15		12-15

**Secondary quotas**			

**Region of origin**	**Male**	**Female**	**Total**
Central Europe^1^	10-12	10-12	20-24
South-Eastern Europe^2^	4-6	4-6	8-12
Northern Baltic^3^	4-6	4-6	8-12
**Sexuality**			
Heterosexual	30-35		30-35
Homosexual	4-6		4-6
**Use of Health Services in the UK for sexual Health reasons**			
Used services	15-25		20-25
Has not used services	15-25		20-25
**Partnership status**			
Currently single (not married, divorced, separated)	At least 5	At least 5	25-30
Currently in a relationship	At least 5	At least 5	10-15

^1^Poland, Lithuania, Czech Republic, Slovakia, Hungary, Slovenia; ^2^Bulgaria, Romania; ^3^Latvia, Estonia

#### Recruitment

When a respondent had completed the questionnaire on the hand-held computer for the community or clinic samples, they were asked whether they would be prepared to participate in further work to explore topics of the questionnaire in greater depth. Information about the in-depth qualitative interviews was then provided in the appropriate language. If interested, respondents were asked to provide their name, phone contact details, and preferred conversational language. They were then contacted by the research team and a short screening questionnaire was completed over the telephone to establish eligibility according to the quota criteria. If eligibility was established, the interviewees' details were passed onto the appropriate interviewer speaking the relevant CEE language who made arrangements for an in-depth interview at a convenient time and location. In most cases the interviews took place on the premises of UCL and when needed interviewees had their travel expenses reimbursed. This helped to ensure that the interviews would take place in a quiet and confidential environment. The interviews could not be linked to the anonymous questionnaire responses.

#### Data collection

Face-to-face semi-structured in-depth interviews were undertaken to develop a contextual understanding of the factors that influence migrants' sexual behaviour, sexual attitudes and health seeking behaviour. Before beginning the interview participants were told the nature and purpose of the study, and assured that their involvement would be confidential and anonymous. They were then asked to sign a consent form and asked permission to tape the interview. Interviews were based on a topic guide which covered personal circumstances, learning about sex, sexual history and relationships, attitudes towards different types of sexual encounters and practices, perceptions of sexual health risks and safer sex, STIs and HIV, contraception and reproductive history, and use of health services. Trained, experienced bilingual qualitative researchers conducted the interviews in the respondent's native language or English, if preferred. Some women asked to be interviewed by a female interviewer.

The interviews lasted 60-90 minutes and were carried as 'conversations with a purpose' [18]. Respondents were offered a £15 high street voucher at the end of the interview as a token of thanks for their time. They were also offered a leaflet containing a list of websites related to sexual health and health services in the UK.

#### Data analysis and sample characteristics

After each interview, the interviewers produced short fieldwork notes containing their reflections on the interview process and summarizing main themes of the interview. The notes were intended to complement the verbal narratives of the interview conversations when carrying out the analysis. The interviews were all digitally recorded and transcribed verbatim. They were then translated into English and analysed using a thematic approach, based on the general principles of 'Framework' analysis [19]. ATLAS.ti qualitative data analysis software http://www.atlasti.com/ was used to code the transcripts [20]. Interviews were anonymised and no identifying data were kept with the transcripts.

The socio-demographic characteristics of the interviewees are presented in Table 3. They included equal proportions of men and women with representatives from all CEE countries excluding Slovenia. They were a relatively young population: more than a quarter were aged 18-24 years and half were aged 25-34 years. Their youth partly accounted for most of them having no dependents, although the majority reported being in a relationship at the time of the interview. Although the group included people who came to the UK before and after EU accession, they were relatively recent arrivals. Around half had lived in the UK for up to 4 years with a maximum stay of 8 years. Almost all of them had good or fluent English language skills. They were relatively well educated: around half had a university degree and the rest had completed at least secondary education. Despite their education and English language proficiency, most of them worked in low-skilled employment sectors such as hospitality, construction or housekeeping. The majority identified as heterosexual and around a quarter identified as gay or bisexual. The majority had registered with a GP.

**Table 3:** Socio-demographic characteristics of the interviewees

		Number of interviewees (total = 40)
**Gender**	Men	21
	Women	19
		
**Region of origin**	Central Europe^1^	22
	South Eastern Europe^2^	11
	Northern Baltic^3^	7
		
**Age**	18-24 years	12
	25-34 years	24
	35-45 years	2
	45+ years	2
		
**Children**	No children	35
	1 or more children	5
		
**Relationship status**	Single	9
	In a relationship	31
		
**Residence in UK**	< 1 year	6
	1-4 years	15
	> 4 years	19
		
**Education**	Secondary/post secondary	22
	University	18
		
**English language proficiency**	Fluent	24
	Good	14
	Poor	2
		
**Occupation**	Student	10
	Hospitality	5
	Construction	3
	Retail	2
	Admin/reception	5
	Cleaning/house-keeping	5
	Security	2
	Other	8
		
**Sexual orientation**	Heterosexual	32
	Gay/bisexual	8
		
**GP registration**	Registered	31
	Not registered	9

^1^Poland, Lithuania, Czech Republic, Slovakia, Hungary, Slovenia; ^2^Bulgaria, Romania; ^3^Latvia, Estonia

## Discussion

One of the strengths of the SALLEE project is in the use of mixed methods to measure and understand sexual and reproductive health among CEE migrants. The study benefits from using both quantitative and qualitative methods, with the cross-sectional survey providing generalisable data on a range of variables and the qualitative research adding in-depth understanding and interpretation. A mixed methods study design is recommended because it produces a more holistic picture of the experiences of a given population than when quantitative and qualitative research are used on their own.

### Quantitative arm

The findings from convenience samples are limited because respondents are not randomly selected to participate. The SALLEE project benefited, however, from using three complementary convenience sampling strategies. The profile of respondents in the three samples is somewhat different because, as with all convenience samples, the composition of the sample is determined by how and where respondents are recruited. The advantage of using a number of sampling strategies is that we can test relationships between the variables in our three different samples which will increase the confidence in our findings.

The community sample was designed to be a representative sample of CEE migrants living in London. In order to collect data in the most cost-effective and efficient manner, we recruited respondents from areas of London where we were most likely to find CEE migrants. We ensured that data were collected from different venues on different times and days but were not able to randomize data collection across times, days and venues because this was not practical with nine part-time fieldworkers with varying commitments over a period of nine months. We recruited 2,276 respondents into our community sample when our original target was 2,000. Given the large sample size, the community sample is likely to provide an adequate representation of CEE migrants who live or use venues in Newham and Hammersmith & Fulham. The generalisability of the findings is limited because respondents were only recruited in two London boroughs. They may not therefore be representative of the population of CEE migrants in London as a whole because other areas may attract different sectors of the CEE migrant population. However, the mapping exercise proved to be very successful in locating CEE migrants. It is recommended for the location of other migrant populations and especially those on whom little or no official data are available.

Convenience surveys are also subject to bias when they are dependent upon fieldworkers approaching study participants. An additional challenge for the SALLEE fieldworkers in the community setting was to identify CEE migrants in the street. Furthermore, some of the potential respondents spoke very little English and it was difficult for fieldworkers to engage them if they did not share the same native language. Fieldworkers were generally more successful in recruiting respondents from their home countries which may also have introduced some bias into the sample, given the imbalance in the nationality of the fieldworkers (4 Polish, 3 Romanian, 1 Bulgarian and 1 Czech) and in the number of shifts that they undertook. While response rates are difficult to measure when recruiting people in the street, our estimated community response rate was low (33.6%) in comparison to estimated response rates of 50-60% in a study which recruited gay men attending London gyms [21]. Although those who refused to take part in our study were estimated to be a little older than those who completed the questionnaire, participation was not associated with gender or the borough where respondents were approached.

In order to address some of the limitations of the community sample, we placed the questionnaire on the Internet so that it could be accessed by anyone from the CEE community living anywhere in London. It should be noted that one of websites used to recruit respondents was particularly successful (a Czech and Slovak community website) and resulted in an over-representation of Czechs and Slovaks in the Internet sample (69.9%). We also used a clinic sample in order to ensure adequate representation of service users and capture people with higher risk behaviour. These clinics are attended by people who live across London, although they are based in North and Central London.

The use of hand-held computers and a web survey for data collection had several advantages for the project. Computerisation enables complex routing of questionnaires without the need for respondents to follow complicated instructions, thereby improving the quality of the data collected [22]. The questionnaire was available in twelve different languages at the click of a button, allowing 92% of respondents to complete it in their native language. The hand-held computers were also extremely practical in the field, both on the streets and in the clinics. They were compact and easy for fieldworkers and respondents to handle, particularly while standing in the street, and more manageable for fieldworkers to administer than pen-and-paper questionnaires in twelve different languages. They were discrete and provided respondents with the privacy to complete the questionnaire, even in busy clinic waiting rooms. Many people were also curious and interested in the hand-held computers and while some of the older people were more cautious about using them, respondents rarely refused to take part because of them. The use of hand-held computers is therefore highly recommended for research of this kind.

Measuring self-reported sexual behaviour is always challenging and especially difficult in a cross-cultural setting. Although piloting failed to reveal any problems, it became apparent that respondents were mis-interpreting one of our key questions during the data collection process. The question, "Have you ever had sexual intercourse with someone of the opposite sex?" was a validated question from Natsal. However, respondents seemed to understand the opposite sex to mean the sex that they do not have sex with ie the same sex, and therefore answered no to the question. This was not related to nationality or the language used to complete the questionnaire. The question was a filter which either opened or closed all subsequent questions on sexual behaviour. We therefore re-routed the questionnaire so that respondents were asked five subsequent questions about sexual behaviour regardless of their response to the opposite sex question. Respondents who answered yes to any of these questions were routed back into the section on sexual behaviour. This problem shows how validated questions may not be transferable from one population to another and thoroughly pre-testing questions using cognitive interviews in cross-cultural work is recommended.

### Qualitative arm

The challenges of conducting research in a cross-cultural environment became especially apparent when organizing qualitative in-depth interviews. In contrast to survey questionnaires, interviews involved sharing one's sexual behaviour and health experiences with the interviewer, which in some cases raised anonymity and confidentiality concerns among participants. When setting up an interview it was important to confirm with the interviewee whether they were happy to be interviewed by a person of the same or opposite sex and by a member of their own ethnic community. In some cases the interviewer's gender was an issue (some women preferred to be interviewed by a woman). Some interviewees felt more comfortable discussing their sexual behaviour in English which they viewed as a 'neutral' language compared to the emotional language of their native country where their sexual behaviour might be frowned upon (especially in the case of gay men). Similarly, some preferred to avoid sharing their experiences with co-ethnic interviewers who they feared may be judgmental of their sexuality or may talk about the interview with other co-nationals. This illustrates the importance of giving interviewees the opportunity to raise such concerns during the initial screening stage. Addressing such issues at this stage makes it possible to overcome some of the barriers to conducting a successful interview. Screening was also important in ensuring that the sample of interviewees reflects a variety of experiences and attitudes.

## Conclusion

The SALLEE project had ambitious aims to conduct a cross-sectional survey and in-depth qualitative interviews with migrants from ten countries in Central and Eastern Europe speaking eleven different languages. It has succeeded in collecting timely data from a representative community sample in the absence of a sampling frame. It will help to illuminate our understanding of the sexual lifestyles, sexual and reproductive health risks and health service needs of these communities and address issues of service planning and the production of appropriate health promotion material.

## Competing interests

The authors declare that they have no competing interests.

## Authors' contributions

FB, GH, CM, CG, RM, RF and JI conceived the study; all authors participated in its design; FB was responsible for the overall project; AE was responsible for the quantitative arm of the study; VP was responsible for the qualitative arm of the study; AE drafted the manuscript with input from FB, VP, GH, CM, CG, RM and RF; all authors read and approved the final manuscript.

## Pre-publication history

The pre-publication history for this paper can be accessed here:

http://www.biomedcentral.com/1471-2458/9/399/prepub
